# Research and practice on key drug safety monitoring under the normalization of centralized procurement: A case study of 13 selected drugs in a Province

**DOI:** 10.1097/MD.0000000000046379

**Published:** 2025-12-12

**Authors:** Guirong Ding, Jinxi Ding

**Affiliations:** a School of International Pharmaceutical Business, China Pharmaceutical University, Nanjing, Jiangsu Province, China.

**Keywords:** adverse drug reactions, drug safety, generic drugs, National Centralized Volume-Based Procurement, real-world data, risk signals

## Abstract

The National Centralized Volume-Based Procurement policy for drugs has effectively reduced drug prices but has raised concerns regarding quality and safety. To address these issues, adverse drug reactions (ADR) are monitored through a national system. As the policy stabilizes, real-world data (RWD) may improve, making ongoing monitoring crucial for studies and assessments. This study monitored 13 drug types from batches 1 to 8 across 39 provincial hospitals, supplied by 12 marketing authorization holders. We compared tracking data with the “National Adverse Drug Reaction Monitoring System” to analyze reporting rates of ADR and identify risk signals for rational drug use in practice. The monitoring period was from May to December 2023, and routine data from 2019 to 2022 were analyzed using descriptive statistics and comparative methods. A safety evaluation of 13 drug types across 39 hospitals revealed ADR rates ranging from 0 to 4.58% and serious reactions ranging from 0 to 1.97%, with 4 identified risk signals. In comparison, routine monitoring indicated lower rates: 0 to 1.20% for ADR and 0 to 0.80% for serious reactions, with one risk signal. Despite these differences, the ADR risks associated with all drugs were manageable, with no unexpected events exceeding label listings. These findings suggest that the safety of domestic generic drugs is comparable to that of originator drugs, supporting the effectiveness of China’s centralized procurement policy. This study aims to enhance coordination among the healthcare, insurance, and pharmaceutical sectors by improving the supervision of drug procurement and RWD research. It emphasizes the need for rigorous monitoring to address safety concerns.

## 1. Introduction

The ongoing issue of excessively high pharmaceutical pricing has long been a significant concern in China’s healthcare system reform.^[1]^ Despite implementing various policies, the public faces challenges such as high medical consultation fees and excessive prescription costs. Since 2015, China has been enacting drug procurement policies to improve and standardize the centralized procurement of pharmaceuticals in public hospitals and medical facilities. This has necessitated a comprehensive revision of the procurement and bidding processes.^[1]^ In 2018, the government identified 4 municipalities directly under its jurisdiction, including Beijing, Shanghai, Chongqing, and Tianjin, along with 7 other cities: Shenyang, Dalian, Xiamen, Guangzhou, Shenzhen, Chengdu, and Xi’an, as pilot cities for the National Centralized Drug Procurement.^[2]^ The core mechanism of volume-based procurement is “quantity for price”; that is, by specifying the purchase volumes, pharmaceutical companies are incentivized to offer lower prices.^[3]^ Statistics show that by the tenth round of National Centralized Drug Procurement since 2018, 435 types of drugs had been successfully procured, with average prices reduced by over 50%.^[4]^ In the tenth round, held in November 2024, 62 drug types were successfully procured, with 385 products from 234 companies selected, achieving an average price reduction of 70%. The significantly reduced prices of many selected drugs have garnered public attention.^[5]^

The significant price reductions in medications have raised public concerns about their quality and safety, further amplified by advancements in information technology, as evidenced by the China Health-Media Group’s public opinion monitoring system, which shows a consistent upward trend in drug-related discussions.^[5,6]^ Notably, in 2023, public opinion data reached over 40 million entries, a decrease of 53.8% from 2022. This decline follows the extensive spread of pharmaceutical and medical device information that intensified between 2020 and 2022 due to the COVID-19 pandemic. However, in 2023, as the focus shifted to treating COVID-19 as endemic, information dissemination decreased sharply compared to 2023, although it remained more than double the levels of 2019.^[7]^ Despite concerns regarding low-priced drugs, peers from clinical and industry fields and patients have emphasized the safety and efficacy of drugs produced by companies with the same generic name.

Adverse drug reactions (ADRs) are harmful or unintended responses to medications administered at standard doses. Therefore, they are a core aspect of drug oversight throughout the life cycle and provide a crucial foundation for post-marketing risk management.^[8]^ In contrast, routine ADR monitoring relies on passive reporting from drug marketing authorization holders (MAHs) and medical institutions. At the same time, key monitoring initiatives can encourage manufacturers to meet their primary responsibilities for quality and safety, improve reporting compliance, and strengthen the safety baseline for drugs acquired through centralized purchasing. However, some companies have automated their production lines to further reduce costs after being included in centralized procurement. Importantly, key monitoring also serves as an effective method for assessing the safety of drugs following price reductions. Nonetheless, this process requires coordination among multiple departments (e.g., medical insurance, public health, and drug regulatory authorities) and significant human resources. As a result, comprehensive monitoring of all drug varieties remains challenging. To address these limitations, our study performed key monitoring on 13 representative drugs in one province, analyzed the results, and identified potential drug safety risks. Ultimately, based on the findings, we propose recommendations to support quality and safety evaluations for centralized procurement of drugs and to enhance regulatory credibility.

## 2. Materials and methods

### 2.1. Sample selection

This research was approved by the Ethics Committee of China Pharmaceutical University. This study was conducted as a retrospective observational analysis. Thirteen drugs included in centralized procurement were selected, such as iodixanol injection. These drugs were supplied by 12 MAHs and used across 39 medical institutions, some of which administered multiple products. Selection of drugs was based on factors such as clinical usage, the magnitude of price reductions, product characteristics, historical adverse reactions, and existing warning signals. The types of research drugs and the number of hospitals using them are displayed in Table 1.

**Table 1:** Research drug varieties and hospital distribution.

No	Drug varieties	Production companies	Medical institutions	Decrease in procurement
1	Paclitaxel for injection (albumin-bound)	1	3	70%
2	Iohexol injection	1	3	55%
3	Iodixanol injection	1	6	72%
4	Iopamidol injection	1	3	67%
5	Oxaliplatin injection	1	8	88%
6	Tigecycline for injection	3	5	67%
7	Clindamycin phosphate injection	1	1	48%
8	Octreotide acetate injection	1	1	26%
9	Canagliflozin tablets	2	3	50%
10	Gemcitabine hydrochloride for injection	2	3	92%
11	Propofol medium/long-chain fat emulsion injection	2	2	80%
12	Esomeprazole sodium for injection	3	1	56%
13	Azacitidine for injection	2	3	70%

For comparative purposes, 3 data sources were used: key monitoring data collected retrospectively from hospital records during May–December 2023; self-monitoring reports submitted by MAHs; and routine monitoring data reported by medical institutions to the National Adverse Drug Reaction Monitoring System between 2019 and 2022.

### 2.2. Data collection

The key monitoring period lasted 6 months, from May to December 2023. Data were retrospectively extracted from hospital information systems, case records, and pharmacovigilance (PV) reports in the participating medical institutions, in accordance with national technical guidance. Collected information included drug utilization, patient demographics, and any suspected ADRs. ADRs were identified through multiple sources, such as physician and pharmacist documentation, patient complaints recorded in medical charts, abnormal laboratory test results, and electronic medical records. All reported ADRs were reviewed and validated by hospital PV teams and subsequently confirmed by the provincial ADR monitoring center. Serious ADRs were classified according to World Health Organization PV criteria, including life-threatening events, hospitalization, disability, or death.

To ensure data integrity, reports from the 39 hospitals and 12 MAHs were consolidated by the research team into a unified dataset. Potential duplicate entries (e.g., the same case submitted by both a hospital and an MAH) were identified by cross-checking patient demographics, hospital admission numbers, ADR occurrence dates, and drug batch numbers. When duplicates were detected, the most complete and consistent version of the record was retained.

In addition, the MAHs collected retrospective ADR reports for the monitored drugs, assessed safety using past data and related information, identified possible risk signals, and implemented necessary risk management measures. In this study, risk signals were defined as newly identified or unusual adverse reaction patterns that exceeded expected background rates described in drug labels, or clinically significant reactions observed in association with specific drug combinations. A signal was considered present when repeated reports or clustering suggested a potential safety concern requiring further evaluation.

Routine monitoring data were obtained from the National Adverse Drug Reaction Monitoring System for the period 2019 to 2022 (4 years) covering 12 drugs. These data were not directly merged with the key monitoring dataset but analyzed separately for comparison, in order to avoid double counting between local reports and national submissions. Routine monitoring results were then integrated with centralized procurement pricing information to assess the relationship between safety monitoring and drug costs. Price data were primarily obtained and processed using the Yaozhi database (https://www.yaozh.com/), which contains pricing information for most pharmaceuticals in China.^[9]^ Additional validation and computation were conducted using data from the National Centralized Volume-Based Procurement Agency. For drugs with multiple manufacturers, the average price reduction was calculated.

### 2.3. Descriptive statistics and analysis

The research assessed the incidence of adverse reactions by quantifying instances of adverse effects, episodes of severe adverse effects, and the number of risk signals (unidentified potential risks). Descriptive statistics and differential analysis were employed. Initially, the prevalence of various types of adverse reactions and the frequency of serious adverse reactions were examined by detailing the conditions of focused and daily monitoring. Subsequently, parametric tests, including the *T*-test and analysis of variance, as well as nonparametric tests such as the Wilcoxon Signed-Rank Test and Kruskal–Wallis Test, were utilized to evaluate the differences between monitoring systems and drug manufacturers.^[10,11]^

## 3. Results

### 3.1. General situation

To avoid bias in readers’ perceptions of specific medical institutions and pharmaceutical companies, this study categorized medical institutions by region and assigned serial numbers to the companies. The key monitoring collected drug use data from 175,899 sales units, while the routine monitoring gathered data from 9,499,401 sales units.

### 3.2. Key monitoring situations

Key monitoring results indicated that the incidence of adverse reactions for 13 drugs ranged from 0.00 to 4.58%. Among these, 6 drugs reported adverse reaction incidences exceeding 0.10%, while the others showed minimal or no adverse reactions. The rate of serious adverse reactions varied from 0.00 to 1.97%. Only 5 drugs demonstrated a serious adverse reaction rate above 0.10% (Table 2), with the remainder showing nearly no serious reactions. Notably, the incidence of adverse reactions for paclitaxel for injection (albumin-bound), oxaliplatin injection, gemcitabine hydrochloride for injection, and azacitidine for injection was higher compared to the other monitored drugs. These 4 drugs are primarily used for treating tumors, leukemia, and similar conditions, and are often administered in combination with other drugs in clinical settings. Given that patients with serious diseases are typically in the second-line and terminal stages of treatment, the probability of experiencing adverse reactions is relatively high, which aligns with the adverse reactions described in the drug labeling.

**Table 2:** Key monitoring status of drug varieties: evaluation of adverse reaction incidence and risk signal detection in a hospital cohort.

Drugs	Hospitals	Patients	ADR	%(A)	SADR	%(S)	RS
Paclitaxel for injection (albumin-bound)	A1	1222	17	1.39	10	0.82	No
	A2	524	54	10.31	26	4.96	No
	A3	282	13	4.61	4	1.42	Yes (1)
Total	**/**	2028	84	4.14	40	1.97	Yes (1)
Iohexol injection	A4	22,457	1	0.00	0	0.00	No
	A5	1680	0	0.00	0	0.00	No
	A6	745	2	0.27	1	0.13	No
Total	**/**	24,882	3	0.01	1	0.00	No
Iodixanol injection	A7	3713	8	0.22	1	0.03	No
	A8	1596	7	0.44	0	0.00	No
	A9	4900	13	0.27	5	0.10	No
	B1	19,695	12	0.06	5	0.03	No
	B2	25,302	11	0.04	2	0.01	No
	B3	1665	5	0.30	3	0.18	No
Total	**/**	56,871	56	0.10	16	0.03	No
Iopamidol injection	A10	1353	2	0.15	0	0.00	No
	A7	21,974	3	0.01	2	0.01	No
	A8	954	1	0.10	0	0.00	No
Total	**/**	24,281	6	0.02	2	0.01	No
Oxaliplatin injection	W1	700	85	12.14	31	4.43	No
	W2	504	28	5.56	10	1.98	Yes
	W3	332	15	4.52	2	0.60	No
	S1	1863	142	7.62	34	1.83	No
	S2	2487	122	4.91	49	1.97	No
	S3	4566	47	1.03	8	0.18	No
	Y1	1344	85	6.32	40	2.98	No
	Y2	245	27	11.02	11	4.49	No
Total	**/**	12,041	551	4.58	185	1.54	Yes
Tigecycline for injection	W4	311	20	6.43	6	1.93	No
	W1	329	0	0.00	0	0.00	No
	W5	194	3	1.55	1	0.52	No
	W3	180	1	0.56	1	0.56	No
	W2	353	4	1.13	1	0.28	No
Total	**/**	1367	28	2.05	9	0.66	No
Clindamycin phosphate injection	H1	4800	2	0.04	0	0.00	Yes (1)
Total	**/**	4800	2	0.04	0	0.00	Yes (1)
Octreotide acetate injection	X1	3027	0	0.00	0	0.00	No
Total	**/**	3027	0	0.00%	0	0.00	No
Canagliflozin tablets	N1	581	2	0.34	0	0.00	No
	N2	1272	0	0.00	0	0.00	No
	N3	1755	1	0.06	0	0.00	No
Total	**/**	3608	3	0.08	0	0.00	No
Gemcitabine hydrochloride for injection	Z1	906	22	2.43	19	2.10	No
	Z2	185	14	7.57	6	3.24	Yes (1)
	Z3	284	4	1.4	1	0.35	No
Total	**/**	1375	40	2.91	26	1.89	Yes (1)
Propofol medium/long-chain fat emulsion injection	T1	976	1	0.10	0	0.00	No
	T2	4302	0	0.00	0	0.00	No
Total	**/**	5278	1	0.02	0	0.00	No
Esomeprazole sodium for injection	Q2	31,560	0	0.00	0	0.00	No
Total	**/**	31,560	0	0.00	0	0.00	No
Azacitidine for injection	Y1	4620	61	1.32	16	0.35	No
	Y2	113	2	1.77	0	0.00	No
	Y3	48	6	12.50	1	2.08	No
Total	**/**	4781	69	1.44%	17	0.36	No

%(A) = percentage of ADR, %(S) = percentage of serious SADR, ADR = adverse drug reaction, Hospitals = medical institutions (de-identified codes), RS = risk signals, SADR = serious adverse reactions.

Additionally, risk signals were identified in 4 drugs. For example, iodixanol injection may lead to dysphonia and a sensation of a foreign body in the throat. In contrast, clindamycin phosphate injection may provoke serious adverse reactions when used with tetanus antitoxin, hormone drugs, or analgesics. All risks associated with adverse reactions are manageable, and no adverse reactions outside the approved labeling have been reported. The frequency of adverse reactions, severe adverse reactions, and risk signal detection for various drug types in a hospital setting is summarized in Table 2.

### 3.3. Routine monitoring

Routine monitoring data indicate that the incidence of adverse reactions to 13 drugs ranged from 0.00 to 1.20%. Among these, the incidence of adverse reactions for 5 drugs was above 0.10%, while the others exhibited minimal or no adverse reactions. The incidence of serious adverse reactions ranged from 0.00 to 0.80% (Table 3). Only 3 drugs had a serious adverse reaction exceeding 0.10%, and the remaining drugs showed few to no occurrences. Only one risk signal was identified through routine monitoring (Table 3). This may be due to the subsequent reporting format, which might have caused medical institutions to overlook the risks that had been addressed.

**Table 3:** Routine monitoring of drug varieties: adverse drug reactions, serious, and risk signals across hospitals.

No	Drug varieties	Hospitals	Patients	ADR	%(A)	SADR	%(S)	RS
1	Paclitaxel for injection (albumin-bound)	1	81,527	307	0.38	112	0.14	Yes (1)
2	Iohexol injection	2	323,784	102	0.03	25	0.01	No
3	Iodixanol injection	2	539,777	570	0.11	84	0.02	No
4	Iopamidol injection	3	242,204	82	0.03	21	0.01	No
5	Oxaliplatin injection	1	81,527	307	0.38	112	0.14	No
6	Tigecycline for injection	4	473,905	188	0.04	66	0.01	No
		5	17,796	3	0.02	0	0.00	No
		3	18,477	13	0.07	5	0.03	No
		8	302,340	58	0.02	24	0.01	No
		9	311,112	83	0.03	15	0.00	No
7	Clindamycin phosphate injection	6	712,800	17	0.00	1	0.00	No
8	Octreotide acetate injection	7	1,366,000	72	0.01	6	0.00	No
9	Canagliflozin tablets	8	81,690	1	0.00	0	0.00	No
		3	3,955,170	4	0.00	0	0.00	No
10	Gemcitabine hydrochloride for injection	9	66,296	796	1.20	533	0.80	No
		3	11,446	34	0.30	24	0.21	No
11	Propofol medium/long-chain fat emulsion injection	2	623,300	8	0.00	0	0.00	No
		10	51,900	0	0.00	0	0.00	No
12	Esomeprazole sodium for injection	2	88,200	4	0.00	0	0.00	No
		11	57,000	0	0.00	0	0.00	No
13	Azacitidine for injection	12	1730	2	0.12	0	0.00	No
		3	91,420	109	0.12	30	0.03	No

%(A) = percentage of adverse reactions, %(S) = percentage of serious adverse reactions, ADR = adverse drug reaction, RS = risk signals, SADR = serious adverse reactions.

### 3.4. Difference analysis

Discrepancies were observed in adverse reaction rates and risk signal monitoring between key and routine monitoring. Additionally, variations may exist in reporting practices among different medical institutions during key monitoring and among pharmaceutical companies during routine monitoring. To investigate these issues further, the following difference analysis method was applied to the collected data.

#### 3.4.1. Differences between key monitoring and routine monitoring

The study identified significant differences in the incidence of ADRs, serious adverse reactions, and the number of risk signals monitored under both key monitoring and routine monitoring. Under routine monitoring, the rates of ADRs and risk signals were significantly lower than those recorded under key tracking. To conduct this analysis, paired variables (key monitoring paired with routine monitoring) were established, and a standard distribution test was performed on these variables. The rank test method was utilized to examine whether substantial differences existed between the 2 monitoring methods for the specified variables. Since the number of paired samples was fewer than 5000, the Shapiro–Wilk (SW) test was applied to the paired variables. The results indicated that the significance levels for each paired variable were below 0.001 (*P* = .001, 0.001, and 0.000, respectively), suggesting that the data were not normally distributed (Table 4). Consequently, the Wilcoxon signed-rank test for paired samples was employed to analyze whether there were differences between the results of key monitoring and routine monitoring for the 13 drugs.

**Table 4:** Shapiro–Wilk test results assess the normality of distribution for key vs routine monitoring.

Paired variables	*Z* value	*P*-value
Incidence of adverse reactions	0.726	.001***
Incidence of serious adverse reactions	0.71	.001***
Number of risk signals	0.533	.000***

*** Represent 1% significance levels.

Furthermore, the Wilcoxon test indicated that the paired variables were significant for all 3 indicators: incidence of adverse reactions, incidence of serious adverse reactions, and the number of risk signals (*P* = .039, 0.037, and 0.083, respectively). The analysis revealed significant differences between the paired variables, with large effect sizes (Cohen *d* values of 0.834, 0.735, and 0.588, respectively). These findings demonstrate significant differences in the incidence of ADRs, serious adverse reactions, and the number of risk signals between the 2 monitoring methods (key monitoring and routine daily monitoring).

### 3.5. Differences in the monitoring of the same product in different medical institutions and enterprises

The overall research data indicated no significant variation in adverse reaction monitoring findings for the same drug, regardless of whether it was under key or routine monitoring across medical institutions and enterprises. This study analyzed 5 of the 13 drug varieties used by more than 5 institutions and produced by over 5 enterprises. Due to the small number of research samples, accurately assessing whether the data conforms to a normal distribution was impossible. Therefore, both parametric and non-parametric tests were employed. The *P*-values from both methods were similar, suggesting that the assumptions (normality, equal variance, etc) were satisfied; consequently, the parametric test results were selected. When the *P*-values differed significantly, it indicated that the assumptions were not met; thus, the non-parametric test results were utilized (Table 5).

**Table 5:** Wilcoxon test results for key vs routine monitoring (ADRs, serious ADRs, risk signals).

Paired variables	*P*-value	Cohen
Incidence of adverse reactions	.039**	0.834
Incidence of serious adverse reactions	.037**	0.735
Number of risk signals	.083*	0.588

ADR = adverse drug reaction.

** , and * represent 5%, and 10% significance levels, respectively.

Table 6 presents the results of parametric (one-way ANOVA) and non-parametric (Kruskal–Wallis test for multiple independent samples) tests. The findings indicate no significant differences in ADR monitoring outcomes for the same drug across various medical institutions and companies (all *P*-values > .1). This demonstrates that the adverse reaction monitoring workflow is standardized across all participating institutions, and the monitoring results are highly reliable.

**Table 6:** Differences in monitoring results between medical institutions and enterprises for the same product.

Monitoring subject	Drug varieties	One-way ANOVA		Kruskal–Wallis test	
		*P* ADR	*P* SADR	*P* ADR	*P* SADR
Medical institutions	Iodixanol injection	.732	.929	.607	.607
	Oxaliplatin injection	.511	.533	.353	.396
	Tigecycline for injection	Non	Non	.406	.406
Enterprise	Tigecycline for injection	Non	Non	.287	.287

“Non” means that due to the small amount of data, all absolute deviations in each cell are constants, and Levene’s *F*-statistic cannot be calculated, so the one-way ANOVA cannot calculate the results.

ADR = adverse reaction rate, SADR = serious adverse reaction rate.

### 3.6. Comparative analysis of adverse reactions with original drugs

The samples exhibiting the highest incidence of general adverse reactions (4.58%) and serious adverse reactions (1.58%) in key monitoring were selected for comparative analysis with adverse reaction data from the originator drugs’ public labels. The general adverse reaction rate for oxaliplatin injection was 4.58% (average across 8 medical institutions), with manifestations primarily including leukopenia, neutropenia, allergic reactions, and thrombocytopenia. These reactions did not exceed those described in the public label for the originator drug (ELOXATIN®, manufacturer: SANOFI-AVENTIS FRANCE) nor surpass the labeled incidence rates of “hematological adverse reactions ≥ 5%” or “all-grade allergic reactions (9–12%).”

The incidence of serious adverse reactions to paclitaxel injection (albumin-bound) was 1.58% (average across 3 medical institutions). The main manifestations included bone marrow suppression and fever. These rates did not exceed those described in the originator drug’s published label (Abraxane®, manufacturer: Abraxis BioScience, LLC) and were lower than the incidence rates specified in the originator drug labels: “The incidence of neutrophil counts below 500/mm^3^ (grade 4) after treatment with this product in European and American patients was 9%, and the incidence of grade 4 neutropenia after treatment with paclitaxel injection was 22%. The incidence of grade 4 neutropenia in Chinese patients after treatment with this product and paclitaxel injection was 7%.”

## 4. Discussion

Since implementing the centralized procurement policy, drug regulatory authorities have closely monitored selected drugs, adhering to “full coverage” and “zero tolerance” principles while ensuring thorough oversight of all enterprises and drug varieties included in the national centralized procurement program. Simultaneously, the medical insurance department has continuously refined procurement policies by mandating reference preparations and high-quality generic drugs that pass quality and efficacy consistency evaluations as minimum requirements, effectively ensuring the quality and safety of procured medications.^[4,12]^

First, research findings indicate that the comprehensive implementation of centralized procurement policies and enhanced supervision by drug regulatory agencies, medical insurance providers, and other relevant departments has standardized ADR monitoring across all participating entities in China.^[13]^ Medical institutions and MAHs have heightened their focus on monitoring adverse reactions associated with the centralized procurement of drugs. Twelve MAHs and thirty-nine medical institutions have established drug vigilance management systems, improving their capability to identify and report suspected adverse reactions, resulting in highly reliable monitoring outcomes.^[2]^ The drug varieties in this study represent key examples that have experienced significant price reductions through centralized procurement. Notably, all serious adverse reactions reported by MAHs include those reported by medical institutions. Furthermore, key monitoring data reveal that the adverse reaction profiles of selected generic drugs neither exceed the ranges specified in product labels nor exhibit significant differences from those of their originator counterparts regarding safety.^[3]^

Second, while routine monitoring relies on spontaneous reporting by drug MAHs, manufacturers, and distributors, evidence confirms that its ability to detect adverse reactions and risk signals is significantly inferior to key monitoring systems.^[14]^ This limitation is partly attributable to underreporting in routine monitoring, which may result from limited awareness, heavy workload, or lack of incentives among healthcare professionals.^[15]^ Although enhanced training and stronger reporting awareness remain important, our findings indicate that these measures alone are insufficient. To strengthen PV, ADR monitoring departments should develop active surveillance strategies, especially for critical or high-risk drugs, to complement routine systems. Such approaches may include sentinel hospital networks, prospective follow-up, or the integration of electronic health records, thereby advancing from basic adverse reaction documentation to fully integrated lifecycle PV operations.^[16]^

Moreover, the observed differences between key monitoring and routine monitoring may not solely reflect true variations in drug safety profiles but also biases introduced by different reporting mechanisms. In particular, the closer supervision and explicit responsibilities in key monitoring may induce a Hawthorne effect, with medical staff becoming more vigilant and proactive in detecting and reporting ADRs. Consequently, discrepancies in ADR incidence and risk signals between the 2 systems should be interpreted with caution, as they may reflect both real-world risks and systematic reporting biases. Future studies should consider quantitative methods, such as capture–recapture models, to better estimate the extent of underreporting in routine monitoring.

To this end, this study also proposes a regulatory direction for regulatory agencies. Regulatory agencies can prioritize key monitoring of products with significant price reductions, which aids regulators in quickly identifying safety issues in drug production linked to price decreases and cost compression. On one hand, relevant companies should proactively conduct research; on the other hand, they should fully leverage the resources of provincial and municipal ADR monitoring centers, sentinel hospitals, and scientific research institutions. For example, active surveillance should be implemented under actual clinical conditions through prospective and retrospective studies to verify risk signals further.

This study has some limitations, primarily the challenges faced in data collection, which have led to a smaller sample size. Due to insufficient data from various types and periods, the study can only reach relevant conclusions through descriptive analysis, overlooking the influence of factors such as drug properties (e.g., the type of disease treated), the external environment (e.g., disease severity, patient condition), and production conditions (e.g., process changes) on ADRs. Future studies will consider these factors to provide more comprehensive research support for regulatory decision-making, medical institution drug use, and patient confidence.

## 5. Conclusion and future perspectives

This study confirms that the National Centralized Volume-Based Procurement policy for drugs has maintained acceptable safety standards, with adverse reaction rates for domestic generics (0–4.58%) comparable to those of originator drugs and without unexpected safety risks. While routine monitoring detected fewer adverse events than active surveillance, all observed risks remained within labeled expectations, supporting the policy’s balance of affordability and safety. Future efforts should prioritize the integration of real-world data and the development of standardized PV protocols across healthcare, insurance, and pharmaceutical sectors to strengthen trust in cost-contained medications.

## Author contributions

**Conceptualization:** Guirong Ding, Jinxi Ding.

**Data curation:** Guirong Ding.

**Formal analysis:** Guirong Ding, Jinxi Ding.

**Funding acquisition:** Jinxi Ding.

**Investigation:** Guirong Ding, Jinxi Ding.

**Methodology:** Guirong Ding, Jinxi Ding.

**Supervision:** Jinxi Ding.

**Validation:** Guirong Ding, Jinxi Ding.

**Visualization:** Jinxi Ding.

**Writing – original draft:** Guirong Ding, Jinxi Ding.

**Writing – review & editing:** Guirong Ding, Jinxi Ding.
